# Mild Electrical Stimulation and Heat Shock Ameliorates Progressive Proteinuria and Renal Inflammation in Mouse Model of Alport Syndrome

**DOI:** 10.1371/journal.pone.0043852

**Published:** 2012-08-24

**Authors:** Tomoaki Koga, Yukari Kai, Ryosuke Fukuda, Saori Morino-Koga, Mary Ann Suico, Kosuke Koyama, Takashi Sato, Tsuyoshi Shuto, Hirofumi Kai

**Affiliations:** 1 Department of Molecular Medicine, Graduate School of Pharmaceutical Sciences and Global COE “Cell Fate Regulation Research and Education Unit”, Kumamoto University, Kumamoto, Japan; 2 Research Fellow of the Japan Society for the Promotion of Science (JSPS), Tokyo, Japan; 3 Department of Medical Biochemistry, Graduate School of Medical Sciences, Kyushu University, Fukuoka, Japan; University of Houston, United States of America

## Abstract

Alport syndrome is a hereditary glomerulopathy with proteinuria and nephritis caused by defects in genes encoding type IV collagen in the glomerular basement membrane. All male and most female patients develop end-stage renal disease. Effective treatment to stop or decelerate the progression of proteinuria and nephritis is still under investigation. Here we showed that combination treatment of mild electrical stress (MES) and heat stress (HS) ameliorated progressive proteinuria and renal injury in mouse model of Alport syndrome. The expressions of kidney injury marker neutrophil gelatinase-associated lipocalin and pro-inflammatory cytokines interleukin-6, tumor necrosis factor-α and interleukin-1β were suppressed by MES+HS treatment. The anti-proteinuric effect of MES+HS treatment is mediated by podocytic activation of phosphatidylinositol 3-OH kinase (PI3K)-Akt and heat shock protein 72 (Hsp72)-dependent pathways *in vitro* and *in vivo*. The anti-inflammatory effect of MES+HS was mediated by glomerular activation of c-jun NH_2_-terminal kinase 1/2 (JNK1/2) and p38-dependent pathways *ex vivo*. Collectively, our studies show that combination treatment of MES and HS confers anti-proteinuric and anti-inflammatory effects on Alport mice likely through the activation of multiple signaling pathways including PI3K-Akt, Hsp72, JNK1/2, and p38 pathways, providing a novel candidate therapeutic strategy to decelerate the progression of patho-phenotypes in Alport syndrome.

## Introduction

Alport syndrome is a hereditary glomerulopathy with progressive proteinuria and nephritis caused by defects in the genes encoding α-3, α-4, or α-5 chains of type IV collagen (COL4A3, COL4A4, COL4A5) in the glomerular basement membrane. Among the hereditary glomerulopathies, X-linked Alport syndrome comprises around 85% of all known cases. Almost all of the Alport syndrome patients develop end-stage renal disease by age 20 to 30 years [1]. To date, various studies have led to the development of potential therapies including angiotensin-converting enzyme inhibitors and aldosterone antagonist among others [2], [3]. However, effective treatment to stop or decelerate the progression of proteinuria and/or nephritis is still unestablished. Proteinuria is not only an indicator for the progression of renal disease, but also a detrimental factor of renal function [4]. In addition, renal inflammation, including the up-regulation of pro-inflammatory cytokines and the infiltration of inflammatory cells, causes tissue injury and fibrosis and subsequently leads to irreversible kidney dysfunction [5], [6]. Therefore, a therapeutic approach of modulating both progressive proteinuria and inflammatory cytokine expression might benefit Alport disease.

Previous studies have shown positive medical benefits in the application of electrical stress in inflammation and wound healing [7]. These effects could be attributed to the fact that mechanical stresses such as shear stress and mild electrical stress can activate several intracellular signaling pathways and subsequently regulate various biological functions [7], [8]. In fact, electrical stress impacts on cellular migration and wound healing by activating phosphatidylinositol 3-OH kinase (PI3K)-Akt [9]. Another modality that affects signal transduction is the application of heat stress, which increases the expression of molecular chaperones, specifically the heat shock proteins (HSPs) [10]. Using a device that simultaneously delivers mild electrical stress (MES) and mild heat stress (HS) *in vitro* and *in vivo* [11], we previously showed that MES+HS treatment ameliorated insulin resistance, suppressed some pro-inflammatory cytokines and decreased fat accumulation in diabetes mouse models by enhancing the insulin-stimulated Akt phosphorylation [12]. We further demonstrated that MES+HS induces the molecular chaperone Hsp72 and protects the liver from hepatic ischemia/reperfusion injury and the stomach from indomethacin-induced gastric ulcer [11], [13]. Kondo, et al. recently reported that MES+HS treatment safely reduced inflammatory markers and tended to decrease kidney injury markers such as serum creatinine and blood urea nitrogen (BUN) in healthy male humans [14]. But despite growing evidence of the beneficial effects of MES+HS treatment, it is uncertain whether MES+HS ameliorates kidney dysfunction such as progressive proteinuria and nephritis.

In the present study, we evaluated the effects of MES+HS treatment on proteinuria and nephritis in mouse model of Alport syndrome. MES+HS significantly ameliorated the progressive proteinuria and renal injury in Alport mice. Accordingly, the expressions of pro-inflammatory cytokines and kidney injury marker were suppressed in MES+HS-treated mice. The anti-proteinuric and anti-inflammatory effects of MES+HS on Alport mice are mediated by the activation of multiple signaling pathways. Our studies thus suggest a novel candidate approach to decelerate the progression of proteinuria and nephritis in Alport syndrome.

## Results

### MES+HS ameliorates progressive proteinuria and renal injury in mouse model of Alport syndrome

**Figure 1:**
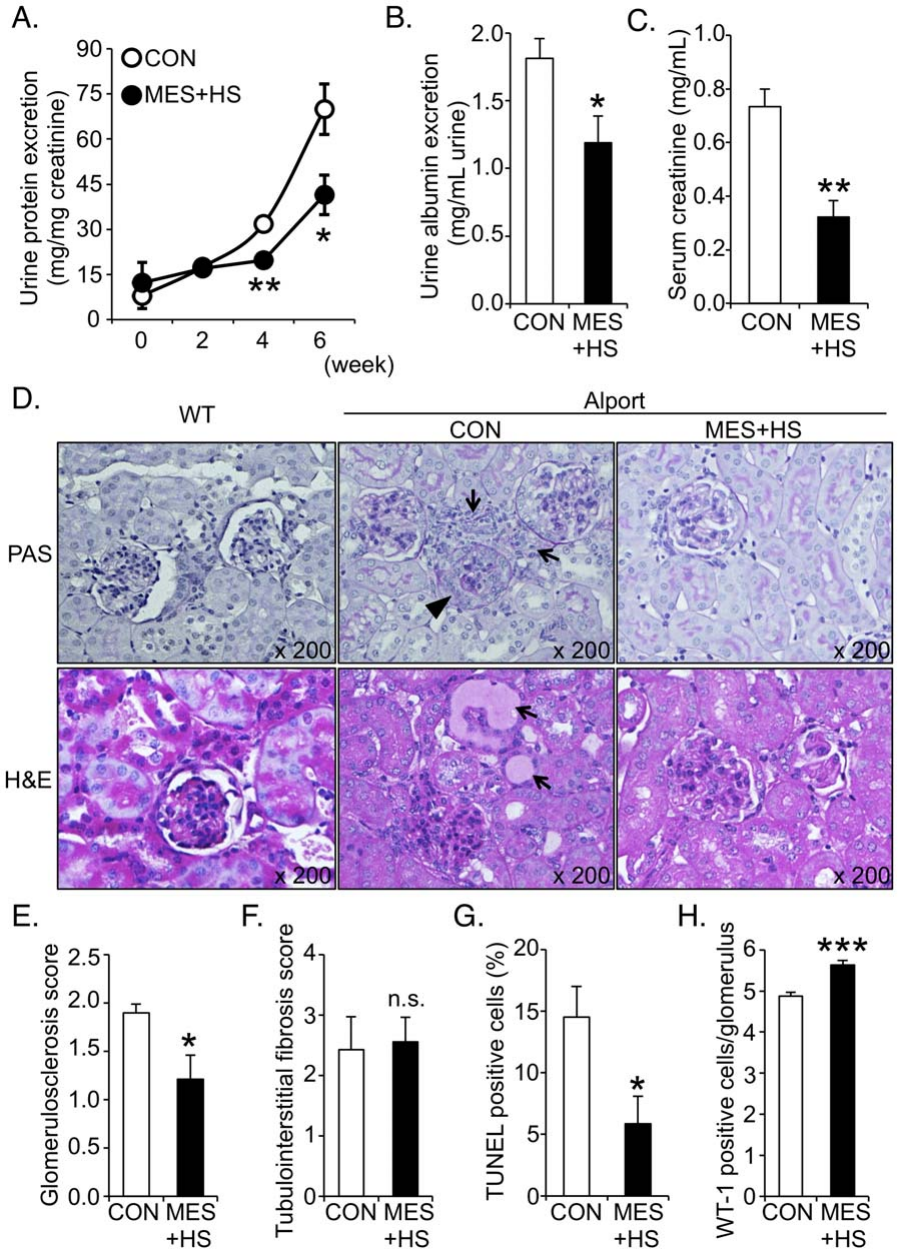
MES+HS ameliorates progressive proteinuria and renal injury in mouse model of Alport syndrome. (*a*) Six- to seven-week-old Alport mice were treated with MES+HS for 10 min twice a week for 6 weeks. Urine samples were collected every 2 weeks. Urinary protein and creatinine were measured by Bradford and Jaffe's method, respectively. Urinary protein concentration was normalized with urinary creatinine concentration. Values are the mean ± S.E. (n = 5 or 6/group). (*b*) Urine albumin excretion was measured in MES+HS-treated and non-treated Alport mice. Values are the mean ± S.E. (n = 6). (*c*) Serum creatinine in sham-treated and MES+HS-treated Alport mice was measured by Jaffe's method. (*d*) Representative images of PAS-stained kidney sections (*upper panels*), and H&E-stained kidney sections (*lower panels*) prepared from MES+HS-treated and sham-treated (CON) Alport mice. *Left panels*, images of age-matched WT mice kidneys are shown. Arrows in upper panels indicates infiltrating inflammatory cells. Arrowhead indicates sclerotic glomerulus. Arrows in lower panels are protein casts. (*e*) Glomerulosclerosis scores were calculated by counting the level of glomerular injury in PAS-stained kidney sections. (*f*) Tubulointerstitial fibrosis scores were evaluated by measuring the region of fibrosis in Masson's trichrome-stained sections. (g) Bar graph shows the average percent of TUNEL-positive cells that were counted per glomerulus. (h) Bar graph shows the average number of WT-1-positive podocytes that were counted per glomerulus immunostained with WT-1 antibody. Values are the mean ± S.E. (n = 5 to 6). *P<0.05, ** *P*<0.01, *** *P*<0.001 assessed by one-way ANOVA with Dunnett's test (*a*) or unpaired *t*-test (*b, c, & e*-*h*).

Congruent with the previous characterization of the established mouse model of X-linked Alport syndrome [15], proteinuria was observed in 24-week-old Alport mice but not in wild type (WT) mice (Fig. S1 *a*). The progress of the disease was marked by worsening proteinuria (Fig. S1 *b*) with accompanying phenotypic changes described previously [15]. To first determine the effect of MES+HS treatment on progressive proteinuria, 6- to 7-week-old male Alport mice were treated with MES+HS for 10 min twice a week for 6 weeks following the protocol described in Methods and the experimental set-up illustrated in Fig. S2 *a*, S2*b*. MES+HS significantly suppressed progressive proteinuria in Alport mice (41.2% and 41.4% suppression at 4 and 6 weeks after starting MES+HS treatment, respectively; Fig. 1*a*). Next, we assessed the urine albumin excretion as an indicator of glomerular injury in MES+HS-treated Alport mice at the 6^th^ week of treatment. MES+HS significantly decreased urine albumin excretion in Alport mice (33.3% suppression; Fig. 1*b*). Moreover, serum creatinine concentration was reduced in mice treated with MES+HS for 6 weeks (52.8% suppression; Fig. 1*c*). Histopathologic abnormalities such as glomerular atrophy (arrowhead) and widespread infiltration of inflammatory cells (arrows, *upper panels*) were improved in MES+HS-treated group (Fig. 1*d*, *right*) compared with sham-treated control (*middle*), indicating the amelioration of renal injury. Moreover, protein casts (arrows, *lower panels*) were rarely observed in MES+HS-treated group compared with sham-treated control (Fig. 1*d*). Age-matched WT kidney sections are also shown (Fig. 1*d*, *left panel* *s*). Consistent with histopathological analysis, glomerulosclerosis score was significantly decreased in MES+HS-treated group, whereas MES+HS did not affect tubulointerstitial fibrosis (Fig. 1*e, 1f* and Fig. S3 *a*). We also assessed the apoptotic cells and podocyte count in the glomeruli. MES+HS reduced the TUNEL-positive cells and increased the Wilm's tumor-1 (WT-1)-positive podocytes in the glomeruli of Alport mice (Fig. 1*g, 1h* and Fig. S3 *b*, S3*c*), suggesting that the treatment inhibited apoptosis. Together, MES+HS treatment ameliorated progressive proteinuria and glomerular injury in Alport mice.

### MES+HS suppresses the expressions of pro-inflammatory cytokines in kidneys of Alport mice in vivo

Despite the fact that nephritis is one of the important patho-phenotypes of Alport syndrome, there is scant information on the expression levels of pro-inflammatory cytokines in X-linked Alport mouse model [5]. So we first compared the inflammation-associated transcriptome in whole kidney RNA of Alport mice and WT mice. Interestingly, pro-inflammatory cytokines such as interleukin-6 (IL-6) and keratinocyte chemoattractant (KC), a potent chemoattractant of neutrophils, were markedly induced at the early stage of Alport disease (8 weeks) compared with WT mice (Fig. S4 *a*, S4*b*) with 12.88-fold and 3.61-fold increase for IL-6 and KC, respectively (Table 1). Tumor necrosis factor-α (TNF-α), interleukin-1β (IL-1β, and tumor growth factor-β (TGF-β) were significantly induced at the middle stage of the disease (16 weeks) in Alport kidneys compared with WT (Fig. S4 *c*-S4*e* and Table 1). Renal injury markers lipocalin-2/NGAL and lysozyme were up-regulated in kidneys of 8-week-old Alport mice that drastically increased as the disease progressed at 16 and 24 weeks (Fig. S4 *f*, S4*g* and Table 1). These data collectively indicated that the pro-inflammatory cytokine gene expression levels are up-regulated in the kidneys of Alport mice compared with WT mice.

**Table 1:** Inflammation-associated transcriptome analysis in kidneys of X-linked Alport syndrome mouse model.

Category	Gene Symbol	Description	Fold change (Alport/WT mouse kidneys)		
			8 week	16 week	24 week
Pro-inflammatory cytokines	IL-6	Interleukin-6	12.88 **	14.4 ***	14.4 **
	KC	Keratinocyte chemoattractant	3.61 **	9.1 ***	20.1 ***
	TNF-α	Tumor necrosis factor-α	1.16 n.s.	2.7 *	4.4 *
	IL-1β	Interleukin-1β	0.83 n.s.	1.6 *	5.8 *
Pro-fibrotic cytokine	TGF-β	Transforming growth factor-β	0.57 **	1.6 *	2.7 *
Renal injury markers	NGAL, Lcn-2	Neutrophil gelatinase-associated lipocalin, Lipocalin-2	14.7 ***	26.0 ***	25.3 ***
	Lyz	Lysozyme	1.90 **	5.1 ***	14.8 **

Total RNA was isolated from kidneys of X-linked Alport mice and age-matched WT mice and subjected to Q-PCR analysis (n = 4 to 5 for 8-week-old mice, n = 6 to 7 for 16- week-old mice, or n = 4 for 24-week-old mice). Fold changes compared to each gene expression in WT mice kidneys are indicated in the Table. *P<0.05, ** *P*<0.01, ***P <0.001 assessed by unpaired *t*-test.

**Figure 2:**
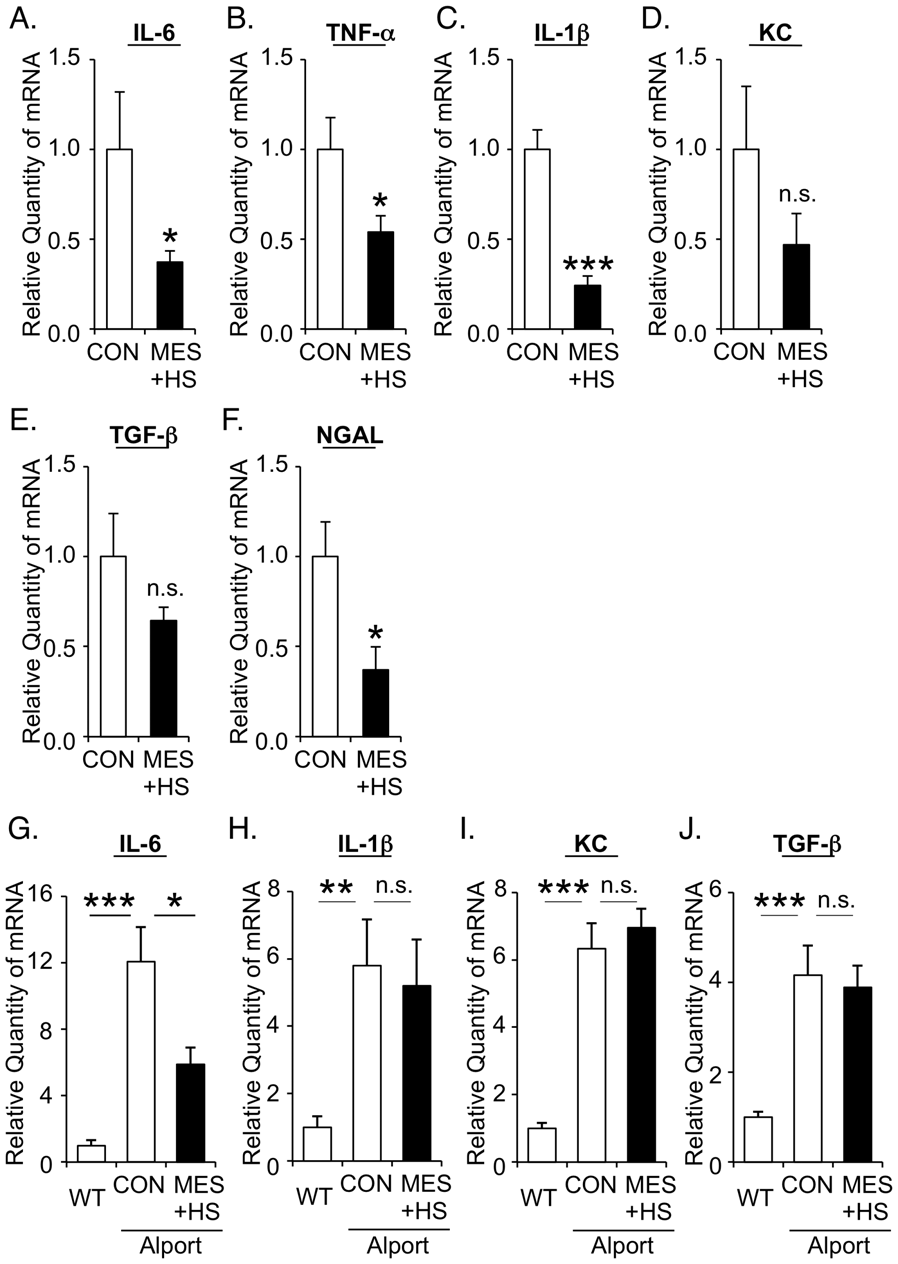
MES+HS suppresses the expressions of pro-inflammatory cytokines in kidneys of Alport mice in vivo. (*a–j*) Forty-eight hours after the last treatment, total RNA was isolated from kidneys (*a*–*f*) or glomeruli (*g*–*j*) of Alport mice sham-treated (CON) or treated with MES+HS for 8 weeks. Q-PCR analysis was performed to assess the expression of the indicated cytokines (*a*–*e, g*–*j*) and renal injury marker NGAL (*f*). Values are the mean ± S.E. (n = 6). *P<0.05, *** *P*<0.001 assessed by unpaired *t*-test. n.s., not significant.

We next investigated the effect of MES+HS on the expression of pro-inflammatory cytokines. Six- to seven-week-old Alport mice were sham treated (control) or treated with MES+HS for 8 weeks. MES+HS treatment significantly suppressed the mRNA expressions of IL-6, TNF-α and IL-1β in kidneys of Alport mice (63%, 46% and 76% suppression *vs* control, respectively; Fig. 2*a*–2*c*). Expression levels of KC and TGF-β were also reduced, albeit not statistically significant (53% and 36% suppression, respectively; Fig. 2*d, 2e*). In addition, the kidney injury marker NGAL was also decreased in MES+HS-treated kidneys (63% suppression; Fig. 2*f*). To evaluate the effect of MES+HS on pro-inflammatory cytokine expression specifically in the glomeruli, Alport mice were treated with MES+HS for 8 weeks, and glomeruli were isolated. For comparison with basal level, we also isolated the glomeruli from WT mice. Quantitative PCR analysis revealed that the expressions of pro-inflammatory and pro-fibrotic genes were up-regulated in the glomeruli of Alport mice compared with WT mice (Fig. 2*g*–2*j*). MES+HS significantly suppressed the expression of pro-inflammatory cytokine IL-6 in the glomeruli of Alport mice (Fig. 2*g*) but not the mRNA expression of IL-1β, KC and TGF-β (Fig. 2*h*–2*j*). Collectively, MES+HS ameliorated renal inflammation by inhibiting the induction of pro-inflammatory cytokines in kidneys and especially the induction of IL-6 in glomeruli of Alport mice.

### MES and HS synergistically activate PI3K-Akt signaling, induce Hsp72 expression and regulate permeability activity in podocytes

**Figure 3:**
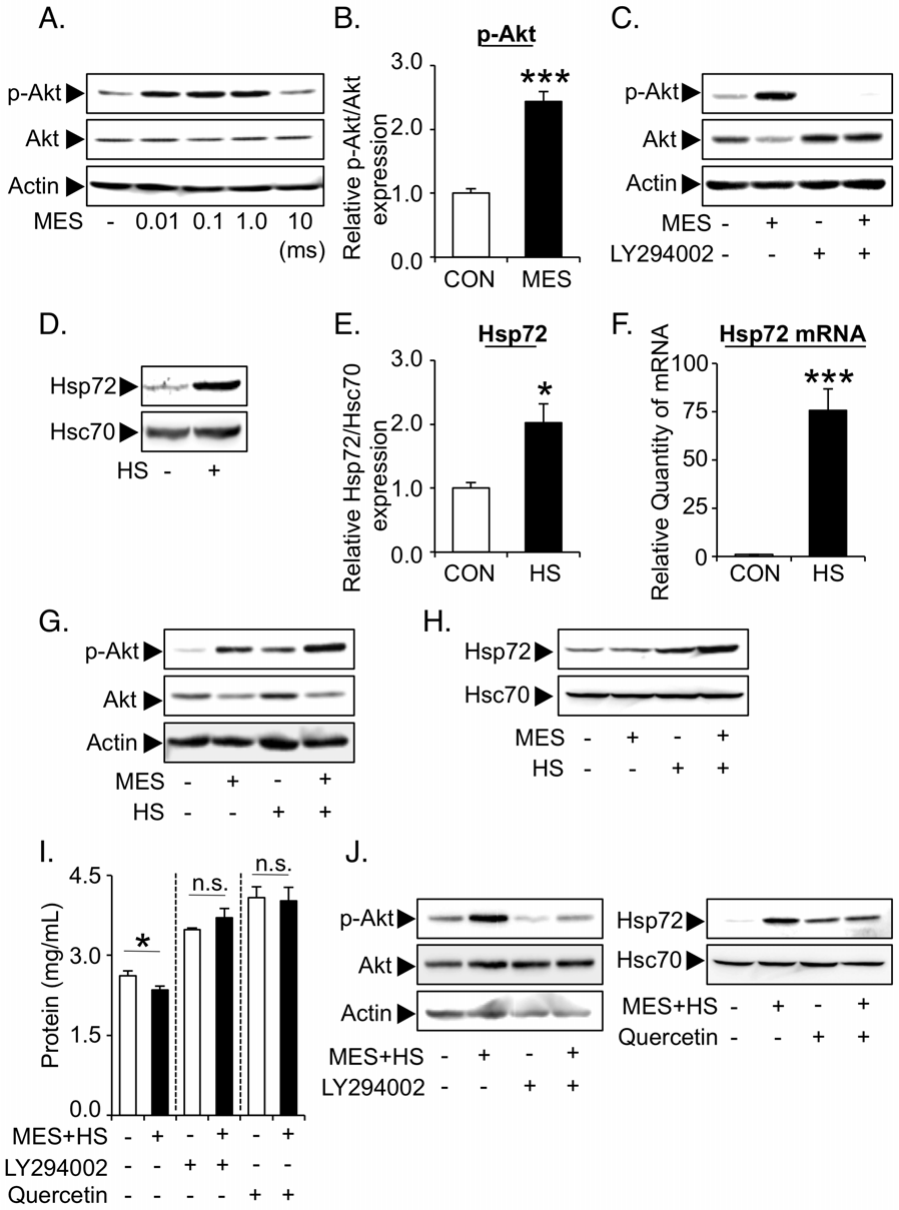
MES and HS synergistically activate PI3K-Akt signaling, induce Hsp72 expression and regulate permeability activity in podocytes. (*a*) Mouse podocytes were treated with MES for 10 min using various pulse durations as indicated. Whole cell proteins were isolated 30 min after MES treatment. Phosphorylated Akt (p-Akt) and total Akt proteins were analyzed by immunoblotting. Actin was used as loading control. (*b*) Relative amount of p-Akt in cells treated with MES (0.1 ms pulse duration) was quantified and normalized to total Akt. (*c*) Cells were treated with 10 µM LY294002 for 1 hr, and stimulated with MES for 10 min. Whole cell protein lysates were isolated after 30 min. (*d*) Whole cell proteins were isolated 5 hr after HS treatment. Hsp72 protein expression was analyzed by Western blotting. (*e*) Relative amount of Hsp72 was quantified with Image Gauge Software and normalized to HSC70. (*f*) Cells were treated with HS for 10 min. Total RNA was isolated after 1 hr and Hsp72 mRNA expression was analyzed by Q-PCR. (*g* & *h*) Cells were co-treated with MES and HS for 10 min. Thirty minutes or 5 hr after the treatment, proteins were isolated and analyzed with p-Akt and total Akt (*g*) or with Hsp72 (*h*) antibodies. (*i* & *j*) Cells were plated onto Transwell chamber and cultured for 8 days. Cells were pre-treated with LY294002 (10 µM) or quercetin (100 µM) for 1 hr and treated with MES+HS for 10 min. (*i*) After treatment, 40 mg/ml BSA in RPMI-1640 solution was added to the bottom chamber. Permeability activity of podocytes was analyzed by measuring the protein concentration in the upper chamber 5 hr after adding the BSA solution. (*j*) Thirty minutes or 5 hr after the treatment, proteins were isolated and immunoblotted respectively with p-Akt and total Akt (*left*) or with Hsp72 (*right*) antibodies. For (*b*, *e*, *f* & *i*), values are the mean ± S.E. (n = 3). *P<0.05, *** *P*<0.001 assessed by unpaired *t*-test. n.s.; not significant.

We next determined the mechanisms of how MES+HS improves various patho-phenotypes in Alport mice. Here we utilized glomerular visceral epithelial cells or podocytes, which are highly specialized cells that regulate the glomerular filtration barrier [16]. It was previously reported that podocytes are cellular origins of COL4A3, 4 and 5 in developing glomeruli, raising the possibility that podocytes could be a therapeutic target in Alport syndrome [5], [17]. Among a variety of regulatory factors for patho-phenotypes of podocytes, PI3K-Akt signaling is considered as one of the most important. PI3K-Akt regulates podocytic survival and foot process reorganization to suppress nephrotic syndrome [18]. Another regulatory factor is Hsp72, which acts as a renoprotective molecular chaperone against various death-inducing stimuli *in vitro* and *in vivo* [19], [20]. We thus examined the effect of MES+HS on Akt and Hsp72 in podocytes. We first evaluated the phosphorylation status of Akt in podocytes after MES treatment. Podocytes were treated with various pulse durations of MES for 10 min as described in Methods section and illustrated in Fig. S2 *c*. Protein lysates were collected 30 min after the treatment and assessed by immunoblotting. MES activated the Akt between 0.01 and 1.0 millisecond (ms) pulse duration in podocytes (Fig. 3*a*). Relative activation of Akt by MES (0.1 ms pulse duration) was 2.5-fold higher in MES-treated group than sham-treated control (Fig. 3*b*). To determine whether Akt activation by MES in podocytes is dependent on PI3K, we inhibited PI3K using a specific inhibitor LY294002. Cells were pre-incubated with 10 µM LY294002 for 1 hr and treated with MES for 10 min. Thirty minutes after MES treatment, protein lysates were collected for analysis. The MES-induced phosphorylation of Akt was mostly inhibited by LY294002 treatment (Fig. 3*c*), suggesting that MES activates Akt mainly through PI3K signaling in podocytes. We next investigated whether HS induces Hsp72 protein expression. Cells were treated with HS at 42°C for 10 min, and protein lysates were extracted 5 hr after treatment. HS induced Hsp72 protein expression more than 2-fold in podocytes (Fig. 3*d, 3e*). The mRNA expression of Hsp72 was also up-regulated in podocytes 1 hr after HS treatment (Fig. 3*f*), indicating that HS increases the transcription of Hsp72. The combination treatment of MES and HS synergistically activated Akt and induced Hsp72 expression (Fig. 3*g, 3h*). Next we assessed the effect of MES+HS treatment on podocyte integrity and filtration barrier function *in vitro* by performing permeability assay as described previously [21]. MES+HS suppressed the leaked protein (Fig. 3*i*). Although the suppression was weak, it was statistically significant. The effect of MES+HS was abrogated by the treatment with LY294002 (10 µM) or with quercetin (100 µM), an HSP inhibitor. The effects of LY294002 and quercetin on Akt activation and Hsp72 induction, respectively, were confirmed by immunoblotting (Fig. 3*j*). These data suggested that MES+HS regulates protein leakage through the activation of PI3K-Akt and the induction of Hsp72 expression in podocyte *in vitro*.

### MES+HS activates Akt and induces Hsp72 expression in podocytes of Alport mice in vivo

**Figure 4:**
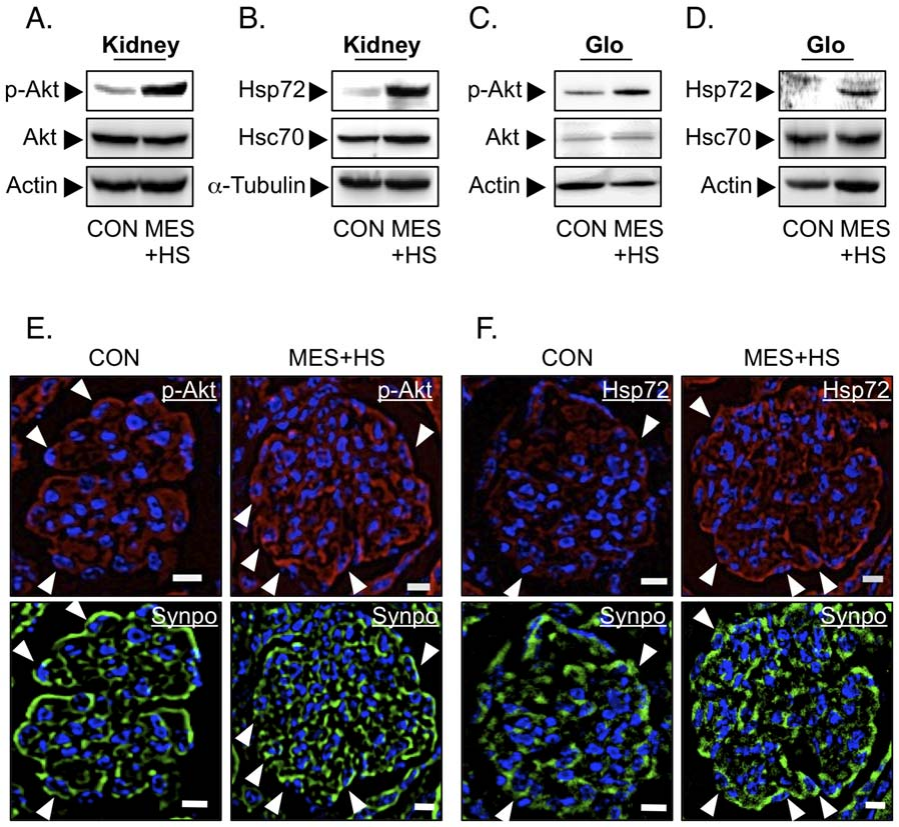
MES+HS activates Akt and induces Hsp72 expression in podocytes of Alport mice in vivo. (*a*, *c & e*) One hour after the final treatment, whole kidney protein lysates or glomeruli protein lysates were isolated from 8 weeks-MES+HS-treated and control Alport mice, and immunoblotted to detect p-Akt and total Akt proteins. Actin was used as loading control (*a, c*). Renal cryosections were subjected to immunohistochemical analysis to assess phosphorylated Akt. Arrowheads indicate phosphorylated Akt in podocytes. Cryosections were counterstained with DAPI (*e*). (*b, d* & *f*) Eight hours (*b* & *f*) or five hours (*d*) after the final treatment, whole kidney or glomeruli protein lysates were isolated from MES+HS-treated (8 weeks treated for *b* & *f*, or 4 weeks treated for *d*) and control Alport mice, and immunoblotted to assess Hsp72 and HSC70 expression. α-Tubulin or actin was used as loading control (*b, d*). Renal cryosections were subjected to immunohistochemical analysis to assess Hsp72. Arrowheads indicate up-regulated Hsp72 in podocytes. Cryosections were counterstained with DAPI (*f*). (*e* & *f*) Representative glomeruli images of three independent experiments are shown. Renal sections were immunostained with anti-synaptopodin antibody to assess podocyte localization (*lower panels*). Sections were counterstained with DAPI. White bars; 10 µm.

We assessed the activation of Akt and induction of Hsp72 *in vivo* by immunoblotting the protein lysates extracted from whole kidneys of sham-treated or MES+HS-treated Alport mice. MES+HS activated the Akt and induced Hsp72 expression in Alport kidneys (Fig. 4*a, 4b*). Moreover, phosphorylated Akt and Hsp72 levels were up-regulated by MES+HS in the glomeruli isolated from treated Alport mice (Fig. 4*c and 4d*). We also examined the effect of MES+HS on Akt activation and Hsp72 induction in podocytes *in vivo* by subjecting MES+HS-treated kidneys to immunohistochemical analysis. Podocytic activation of Akt and induction of Hsp72 expression were seen in kidneys of MES+HS-treated Alport mice (Fig. 4*e, 4f*, arrowheads). Localization of podocytes was determined by double immunostaining with anti-Synaptopodin antibody (Fig. 4*e, 4f*). Collectively, these data suggested that MES+HS ameliorates progressive proteinuria likely via podocytic activation of Akt and induction of Hsp72 expression in Alport mice.

### MES+HS directly suppresses the expressions of pro-inflammatory cytokines in isolated glomeruli of Alport mice ex vivo

**Figure 5:**
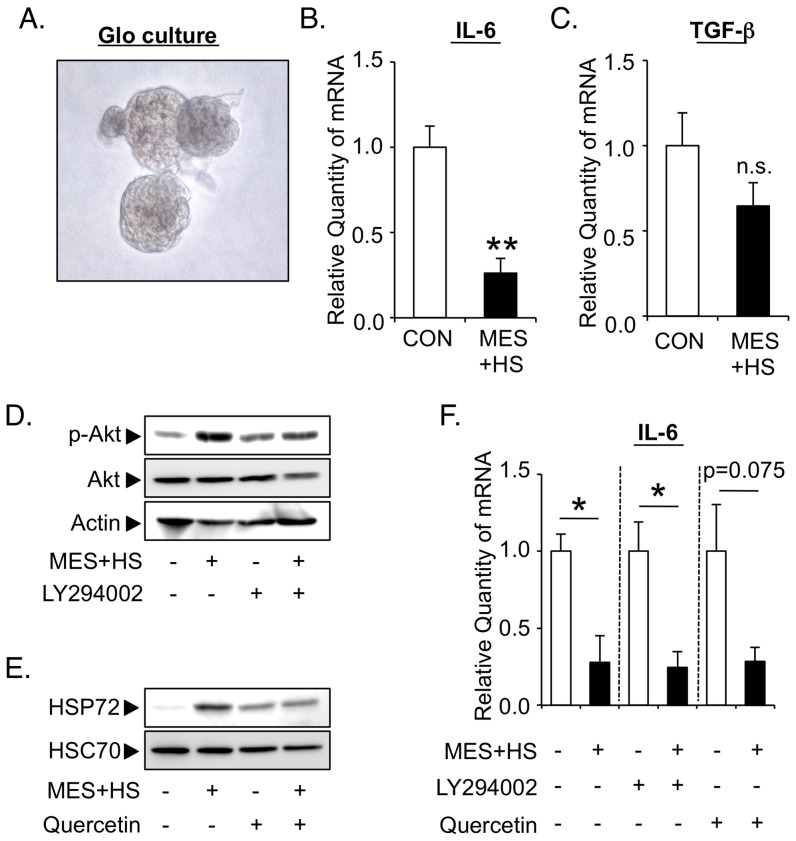
MES+HS directly suppresses the expressions of pro-inflammatory cytokines via PI3K-Akt or Hsp72-independent pathway in isolated glomeruli of Alport mice ex vivo. (*a*) Bright field image indicates isolated glomeruli from Alport mice in culture medium. (*b, c*) Glomeruli were isolated from Alport mice by mesh sieving followed by *ex vivo* MES+HS treatment for 10 min. Four hours after treatment, total RNA was extracted from glomeruli and subjected to Q-PCR analysis to assess the cytokines mRNA expression. (*d*–*f*) Isolated glomeruli from Alport mice were pre-treated with LY294002 (20 µM) or quercetin (200 µM) for 2 hr, and treated with MES+HS for 10 min. Thirty minutes after the treatment, proteins were isolated, and immunoblotted with p-Akt and total Akt antibodies (*d*). Five hours after the treatment, protein lysates were analyzed for Hsp72 expression (*e*), or total RNA was isolated and subjected to Q-PCR analysis to assess the expression of IL-6 (*f*). Values are the mean ± S.E. (n = 3 for b, *c* & *f*). *P<0.05, **P<0.01 assessed by unpaired *t*-test. n.s., not significant.

Because MES+HS suppressed pro-inflammatory cytokines in kidneys of Alport mice *in vivo* (Figure 2), we next determined whether the anti-inflammatory effect of MES+HS is a direct or a subsequent effect derived from its anti-proteinuric function (Fig. 1*a*–*c*). We first isolated glomeruli from 8-week-old Alport mice by steel mesh sieving method and cultured the glomeruli *ex vivo* (Fig. 5*a*). Cultured glomeruli were treated with MES+HS for 10 min. Four hours after treatment, RNA was extracted for analysis of pro-inflammatory and pro-fibrotic genes known to correlate with glomerular disease [5], [22]. Interestingly, IL-6 was significantly suppressed by MES+HS in isolated glomeruli of Alport mice (74% suppression; Fig. 5*b*). Expression of TGF-β were somewhat reduced although this reduction was not statistically significant (Fig. 5*c*). Taken together, the decrease of pro-inflammatory cytokines expression induced by MES+HS was not secondary to the anti-proteinuric effect, but rather a direct effect of MES+HS on cytokines.

### MES+HS suppresses pro-inflammatory cytokines through the activation of JNK1/2 but not through the activation of PI3K-Akt or Hsp72-dependent pathway in Alport glomeruli ex vivo

**Figure 6:**
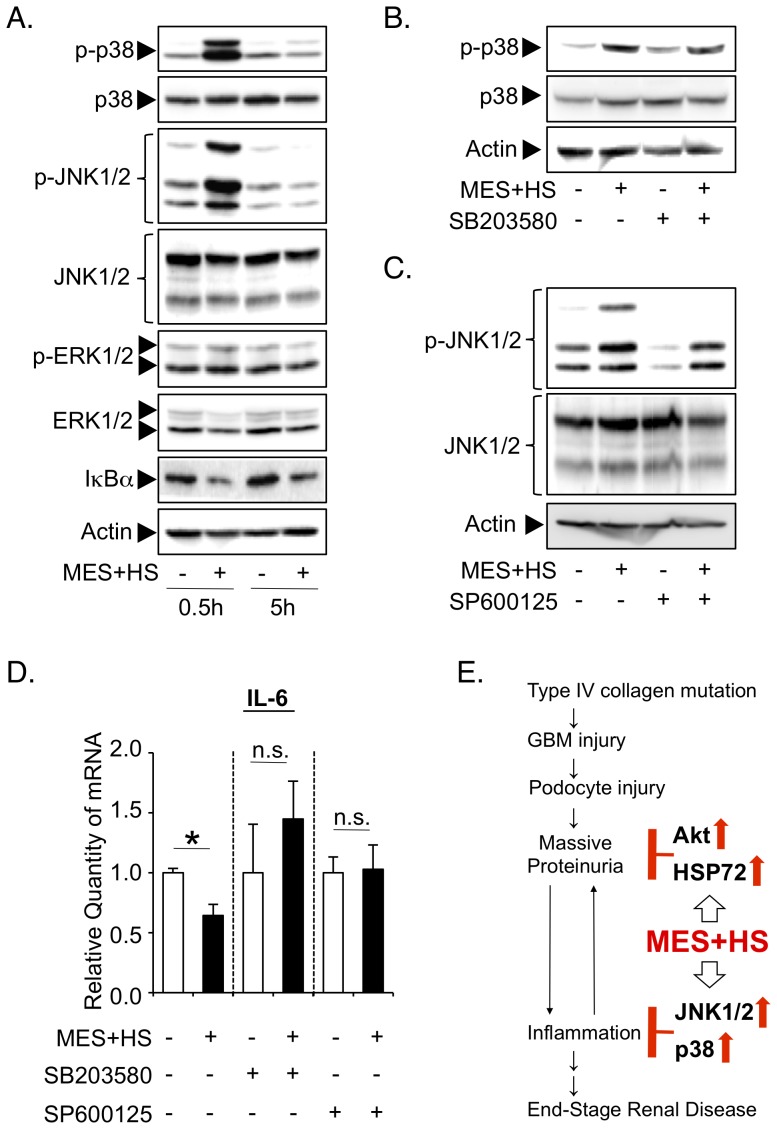
MES+HS suppresses pro-inflammatory cytokines mainly via p38 and JNK1/2 activation in Alport glomeruli ex vivo. (*a*) Isolated glomeruli from Alport mice were treated with MES+HS for 10 min. Thirty minutes or 5 hours after the treatment, proteins were isolated, and immunoblotted with the indicated antibodies. (*b*–*d*) Alport glomeruli were pre-treated with SB203580 (20 µM) or SP600125 (20 µM) for 2 hr and treated with MES+HS for 10 min. (*b* & *c*) Thirty minutes after the treatment, proteins were isolated, and subjected to Western blot analysis with the indicated antibodies. (*d*) Five hours after the treatment, total RNA was isolated and subjected to Q-PCR analysis to assess the expression of IL-6. Values are the mean ± S.E. (n = 3 for *d*). *P<0.05 assessed by unpaired *t*-test. (*e*) Schematic diagram of how MES+HS ameliorates progressive proteinuria and nephritis in Alport syndrome.

To determine the mechanistic details of the anti-inflammatory effect of MES+HS, we first assessed the involvement of Akt and Hsp72. Although pre-treatment with LY294002 or quercetin abrogated the MES+HS-induced activation of Akt or increase of Hsp72, respectively (Fig. 5*d, 5e*), these inhibitors did not affect the MES+HS-induced down-regulation of pro-inflammatory cytokine IL-6 (Fig. 5*f*). These data suggested that MES+HS suppresses cytokine expression independently of PI3K-Akt or Hsp72 pathways. Because mitogen-activated protein kinases (MAPKs) and NF-κB signaling pathways are known to regulate the expressions of inflammatory cytokines [23], [24], we analyzed the activation status of MAPKs p38, c-jun NH_2_ terminal kinase 1/2 (JNK1/2) and extracellular-regulated kinase 1/2 (ERK1/2), and NF-κB in MES+HS-treated Alport glomeruli *ex vivo*. p38 and JNK1/2, but not ERK1/2, were phosphorylated in glomeruli 30 min after MES+HS treatment (Fig. 6*a*). Degradation of IκBα, indicating NF-κB pathway activation was also observed in glomeruli at 30 min and 5 hr after the treatment (Fig. 6*a*). Of these activated pathways, p38 and JNK1/2 are known to negatively regulate the expressions of pro-inflammatory cytokines [25], [26]. We thus evaluated the involvement of p38 and JNK1/2 in the effect MES+HS on pro-inflammatory cytokines in Alport glomeruli *ex vivo* by using specific inhibitors, SB203580 for p38 and SP600125 for JNK1/2. These inhibitors respectively blocked the MES+HS-induced activation of p38 and JNK1/2 (Fig. 6*b, 6c*). Importantly, MES+HS-induced suppression of IL-6 was markedly abrogated by inhibition of p38 and JNK1/2 (Fig. 6*d*). These data suggested the predominant involvement of p38 and JNK1/2 in cytokine suppression by MES+HS in isolated Alport glomeruli *ex vivo*.

## Discussion

In Alport syndrome, mutations of type IV collagen cause glomerular basement membrane (GBM) injury and subsequent podocyte injuries that lead to a vicious cycle of massive proteinuria and renal inflammation, and finally cause end-stage renal disease (Fig. 6*e*). In this study, we have shown that MES+HS treatment activated multiple signaling pathways that ameliorated two important patho-phenotypes in Alport syndrome - proteinuria and inflammation (Fig. 6*e*). Podocytic activation of PI3K-Akt signaling pathway and induction of Hsp72 expression by MES+HS improved the proteinuria *in vitro* and *in vivo* and renal injury in Alport mice. Hsp72 protects renal epithelial cells from acute lethal injury and also ameliorates sub-lethal injury by preventing cytoskeletal collapse, by improving the function of cell-cell junction and by enhancing cell-matrix interaction [27], [28]. It is therefore not surprising that the induction of Hsp72 by MES+HS conferred some protection against renal injury and proteinuria (Fig. 1, 3 & 4). As for the activation of Akt by MES+HS (Fig. 3*g*), we previously reported that MES+HS enhances the phosphorylation of insulin receptor and activates PI3-Akt signaling [12]. The important role of insulin receptor for podocytic function was recently highlighted by the study of Welsh, et al. [29]. On the other hand, Akt is a critical downstream molecule in nephrin-PI3K signaling in podocytes for actin cytoskeleton reorganization [18]. Thus, we cannot eliminate the possibility that MES+HS activates PI3K-Akt signaling via nephrin. The functional activation of insulin receptor and nephrin requires their recruitment to lipid rafts [30], [31]. Consistent with these studies, treatment with methyl-β-cyclodextrin, which depletes membrane cholesterol and ablate lipid rafts, eliminated the MES+HS-induced activation of Akt in podocytes (Fig. S5 *a*). Whether the insulin receptor or nephrin mediates the MES+HS-induced activation of Akt remains to be clarified, but we have shown here that the PI3K-Akt pathway is involved in the anti-proteinuric effect of MES+HS.

Recent reports showed that pro-inflammatory cytokines, chemokines and their receptors are attractive therapeutic targets of Alport syndrome [32], [33], [34]. Podocytic IL-6 induction is thought to constitute a maladaptive phenotype such as the up-regulation of matrix metalloproteinase-3 and -10 (MMP-3 and -10) that degrades the GBM. Thus, inhibition of pro-inflammatory cytokine IL-6 has become a potential therapeutic strategy for Alport syndrome [5]. We found that IL-6 was up-regulated in the early stage of Alport disease in mice (Fig. S4 *a* and Table 1) and that MES+HS suppressed IL-6 in Alport mouse glomeruli *ex vivo*, predominantly through the activation of p38 and JNK1/2 signaling pathways. Inhibition of these pathways interfered with the anti-inflammatory effect of MES+HS. How MES+HS activates JNK1/2 and p38 in the glomeruli (while ERK1/2 was not activated) is still unclear. JNK1/2 and p38 are primarily activated by stress-induced stimuli, hence they are known as stress-activated MAPKs, while ERK is primarily activated in response to hormones and growth factors [23]. Therefore, it might be possible that MES+HS acts as a stimulus to activate JNK1/2 and p38 pathways. Although JNK1/2 and p38 MAPKs are mostly known as mediators of inflammation, Kietzmann, et al. demonstrated that JNK1/2 and p38 signaling pathways could induce an anti-inflammatory molecule, heme oxygenase-1 (HO-1) likely through different transcription factors [35]. Consistent with this report, HO-1 is up-regulated by MES+HS in glomeruli (Fig. S5 *b*). Degradation of IκB usually leads to nuclear translocation of p65 and subsequent transcriptional activation of inflammatory genes in the NF-κB pathway. However, a recent report showed that glutathionylation of p65 after IκBα degradation inhibits its translocation and that the glutathionylation of p65 is regulated by HO-1 [36]. While degradation of IκBα was observed in MES+HS-treated glomeruli (Fig. 6*a*), MES+HS was able to suppress pro-inflammatory cytokine expression in isolated glomeruli (Fig. 5*b, 5f*, 6*d*). Based on these findings, we hypothesized that HO-1 induced by MES+HS leads to glutathionylation of p65 and inhibition of its nuclear translocation, thus suppressing the induction of pro-inflammatory genes.

Of particular interest in this study was that MES+HS treatment tended to prolong survival of Alport mice until around 170 days. However, beyond this time point, MES+HS treatment did not extend the lifespan of Alport mice as assessed by Kaplan-Meier method (Fig. S6). Although MES+HS treatment did not improve the survival of Alport mice, it clearly exerted anti-inflammatory and anti-proteinuric effects. Thus, its beneficial point is its ability to ameliorate the severity of the phenotype. From early to middle stages and before the onset of the late stage, MES+HS treatment is able to lessen the degree of glomerular pathologies associated with Alport disease. Preserving the GBM/extracellular matrix integrity before the onset of proteinuria leads to significant disease protection, but once this opportunity has gone, MMP-inhibition at the later stages of Alport syndrome leads to rather accelerated glomerular and interstitial fibrosis [37]. Importantly, IL-6 has been known to regulate the expression of MMPs in podocytes [5]. Because MES+HS treatment significantly inhibited IL-6 expression (Fig. 2, 5, 6), future investigation will focus on the effect of MES+HS on MMPs especially at different stages of Alport disease in order to optimize the timing and condition of treatment. Based on the findings of our study and our previous report that MES+HS treatment effectively inhibited chronic inflammation in healthy male humans without perceived adverse effects or muscle contraction/pain [14], MES+HS provides a novel, possible approach to decelerate the progression of proteinuria and renal inflammation in Alport syndrome.

## Materials and Methods

### Reagents and Antibodies

LY294002, quercetin and SB203580 were from Sigma. SP600125 was from BIOMOL. Antibodies against phospho-Akt (Ser473), Akt, phospho-p38 (Thr180/Tyr182), p38, phospho-JNK1/2 (Thr183/Tyr185), JNK1/2, phospho-ERK1/2 (Thr202/Tyr204), ERK1/2 and IκBα were from Cell Signaling Technology. Hsp72 and HSC70 antibodies were from Stressgen Biotechnologies. Synaptopodin antibody was from PROGEN. Actin and α-Tubulin antibodies were from Santa Cruz Biotechnologies. HRP-conjugated secondary antibodies were from Jackson Immuno Research Laboratories. Alexa 488-conjugated or 546-conjugated secondary antibody was from Invitrogen.

### Cell culture, isolated glomeruli culture, in vitro MES+HS treatment and Permeability assay

Mouse podocyte cell line MPC5 was kindly provided by Dr. Peter Mundel, and cells were maintained as described previously [38], [39]. Glomeruli were isolated from kidneys of Alport mice by using metal sieves (180, 100 and 71 µm pore size) and cultured with RPMI-1640 supplemented with 10% fetal bovine serum (FBS) and antibiotics. Cells and glomeruli suspension were plated on 60-mm culture dishes and treated with MES and/or HS for 10 min as described previously [12], [40]. Briefly, MES at 1 V/cm and 0.1 millisecond (ms) pulse duration was applied. For HS, culture plates were sealed and immersed in water bath at 42°C or 37°C (for control). For MES+HS, cells were treated simultaneously with MES and HS for 10 min. Podocytic permeability activity was analyzed as described previously [21].

### Animals and in vivo MES+HS treatment

X-linked Alport syndrome mouse model (*Col4a5* ^tm1Yseg^ G5X mutant) was described previously [15]. Mice were obtained from the Jackson Laboratories (Bar Harbor, Maine) and fed with food and water *ad libitum*. Mice were bred as homozygotes by crossing with homozygous females and hemizygous males, and age-matched C57BL/6 mice (Charles River Laboratories Inc.) were used as WT controls. Six- to seven-week-old male mice were treated with MES+HS for 10 min twice a week as described previously [12], [13]. Briefly, mice were placed into well-ventilated chamber and MES was applied to unanesthetized mice through electro-conductive and thermo-generative rubber electrodes with moist soft cotton cloths connected to Biometronome™ (Tsuchiya Gum Co., Ltd.). The condition of MES was 3 V/cm (55 pps) of direct current with individual pulse duration of 0.1 ms. For HS, temperature at the surface of electrodes was adjusted to 42°C. All animal experiments were approved by the Animal care and Use committee of Kumamoto University (#Q21–302).

### Western blot analysis

Cells and tissues were lysed in lysis buffer and subjected to SDS-PAGE and Western blot analysis as described previously [12]. Blots were reacted with specific primary antibodies and with respective HRP-conjugated secondary antibodies. SuperSignal WestPico chemiluminescence substrate (Thermo Scientific Inc.) was used for visualizing the blots.

### Real-time Quantitative RT-PCR Analysis (Q-PCR)

Total RNA was isolated with RNAiso reagent (Takara Bio Inc.) following the manufacturer's instructions. Reverse transcription and PCR amplifications were performed as described previously [13]. The sequences of primers used for Q-PCR are listed in Table 2. The primer sequences for mouse IL-6, TNF-α and IL-1β were described previously [13].

**Table 2:** Primer sequences for Real-time quantitative RT-PCR.

Gene	Sense	Antisense
mouse Hsp72 (HspA1)	5′-­-GATCACCATCACCAACGAC-­-3′	5′-­-ACTTGTCCAGCACCCTTCTTC-­-3′
KC	5′-­-TGTCAGTGCCTGCAGACCAT-­-3′	5′-­-GAGCCTTAGTTTGGACAGGATCTG-­-3′
mouse TGF-β1	5′-­-CACCTGCAAGACCATCGACAT-­-3′	5′-­-GAGCCTTAGTTTGGACAGGATCTG-­-3′
mouse NGAL	5′-­-CAGAAGGCAGCTTTACGATG-­-3′	5′-­-CCTGGAGCTTGGAACAAATG-­-3′
mouse lysozyme	5′-­-CCAGTGTCACGAGGCATTCA-­-3′	5′-­-TGATAACAGGCTCATCTGTCTCA-­-3′

### Evaluation of albuminuria, proteinuria and creatinine concentration

Urine samples were subjected to 12% SDS-PAGE and gels were stained with CBB solution. Densitometric analysis was performed by Image Gauge software and the data were normalized with the image density of 1 µg BSA standard. Urinary protein and creatinine were also measured by Bradford method (Bio-Rad Laboratories) and Jaffe's method (Wako Pure Chemical Industries), respectively. Urinary protein concentration was normalized with urinary creatinine concentration, and presented as urine protein excretion.

### Evaluation of glomerulosclerosis score and tubulointerstitial score

Glomerulosclerosis score and tubulointerstitial score were evaluated as described previously with minor modifications [41], [42]. Briefly, for glomerulosclerosis score, PAS-stained 50–70 random glomeruli per mouse were evaluated and scored from 0 to 4: 0, no lesion; 1, expansion of mesangial area; 2, expansion of Bowman's epithelial cells, and adhesion of glomeruli and Bowman's capsule; 3, sclerotic area: 50 to 75% of glomerulus; 4, sclerotic area: 75 to 100% of glomerulus. For tubulointerstitial score, Masson's Trichrome (MT)-stained 25 to 30 random fields per sample were evaluated by calculating MT-stained area *vs* unstained area as percentages with ImageJ software. Mean scores were obtained and calculated (n = 6).

### Renal Histology and immunohistochemistry

For renal histology, mouse kidneys were fixed in 10% formalin and embedded in paraffin for H&E or PAS staining in O.C.T. compound for immunohistochemistry. Tissue blocks were sliced into 6-µm thickness by using cryostat. Representative sections were stained with H&E or PAS for histological examinations. For immunohistochemistry, the slides were treated with cold acetone for 5 min and immunostained with anti-WT-1, anti-phospho-Akt, anti-Hsp72 or anti-Synaptopodin antibodies. Sections were counterstained with DAPI solution (1 µg/mL). For podocyte counting, 30–50 random glomeruli per mouse were selected and Wilms' tumor-1 (WT-1)-positive cells per glomerulus were counted. Mean scores were obtained and calculated. (n = 5; sham-treated control group, n = 6; MES+HS-treated group).

### TUNEL staining

Glomerular apoptosis was evaluated by staining paraffin section with the Dead End Fluorometric TdT-mediated dUTP Nick-End Labeling (TUNEL) System (Promega). For assessment of TUNEL positive cells, only cells of the glomerular tuft were scored. TUNEL-positive cells were counted by fluorescent microscopy in more than 50 glomeruli per mouse. Sections were counterstained with DAPI solution (1 µg/mL). Mean scores were calculated and expressed as percentage. (n = 6).

### Statistical analysis

Data are presented as mean ± S.E. Significance of the difference between groups were assessed with Student's unpaired two-tailed *t*-test (when 2 groups were analyzed) or one-way ANOVA (for more than 3 group). P value less than 0.05 was considered as statistically significant.

## Supporting Information

Figure S1 **Alport mice exhibit progressive proteinuria in an age-dependent manner.**
(PDF)

Figure S2 **Diagrams of MES+HS treatment in vivo and in vitro and the experimental design.**
(PDF)

Figure S3 **MES+HS ameliorates glomerular apoptosis but has minimal effect on tubulointerstitial fibrosis.**
(PDF)

Figure S4 **Inflammation-associated transcriptome analysis in kidneys of X-linked Alport syndrome mouse model.**
(PDF)

Figure S5 **MES+HS activates Akt through membrane cholesterol in podocyte in vitro and induces HO-1 mRNA expression in Alport glomeruli ex vivo.**
(PDF)

Figure S6 **MES+HS does not affect the survival of Alport mice.**
(PDF)
