# Supplementary material for: Mild Electrical Stimulation and Heat Shock Ameliorates Progressive Proteinuria and Renal Inflammation in Mouse Model of Alport Syndrome
Source: PLoS One. 2012 Aug 24;7(8):e43852. doi: 10.1371/journal.pone.0043852 (PMC3427222; doi:10.1371/journal.pone.0043852)
Supplement: Figure S1 — Alport mice exhibit progressive proteinuria in an age-dependent manner. (PDF) [file pone.0043852.s001.pdf]

Figure S1.

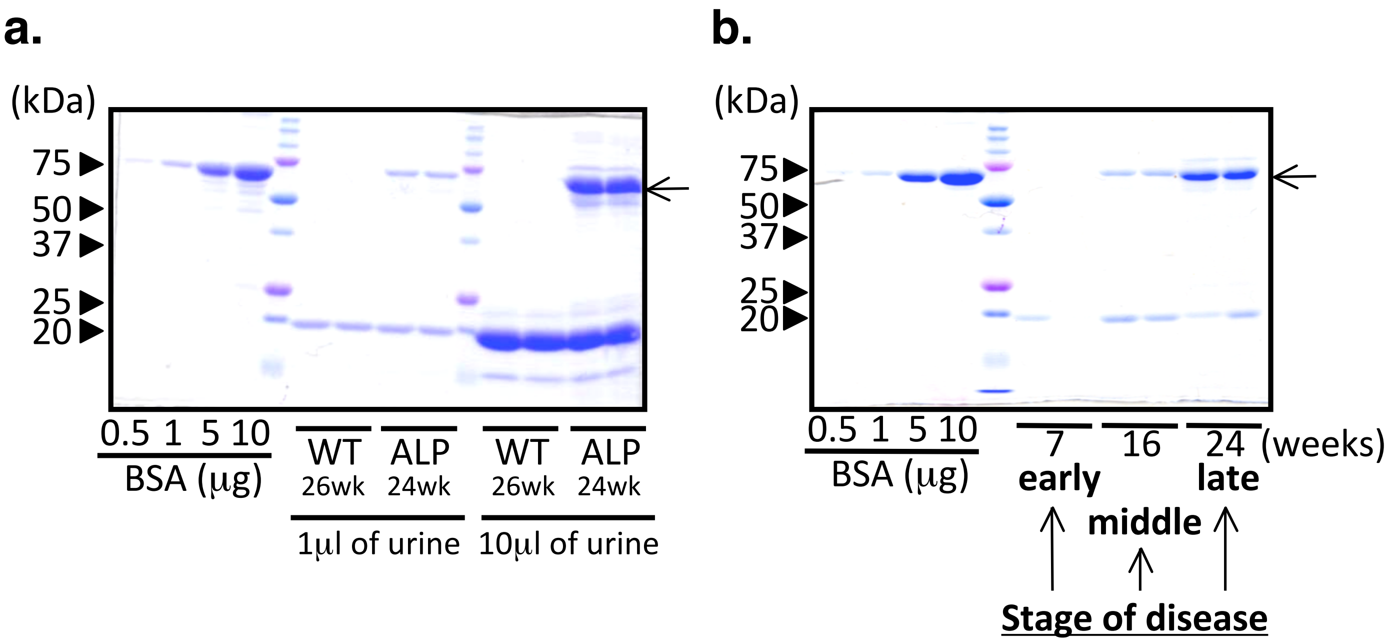

Figure S1. ***Alport mice exhibit progressive proteinuria in an age-dependent manner.*** (*a*) Urinary protein excretion was assessed by 12% SDS-PAGE followed by CBB staining. Urine samples were collected from 26-week-old (wk) WT or 24-week-old (wk) male Alport mice (ALP). (*b*) Urinary protein excretion was gradually dysregulated in Alport mice. Urine samples were collected from 7-, 16-, or 24-week-old Alport mice. Arrows indicate urine albumin. BSA: bovine serum albumin.
