# Supplementary material for: Mild Electrical Stimulation and Heat Shock Ameliorates Progressive Proteinuria and Renal Inflammation in Mouse Model of Alport Syndrome
Source: PLoS One. 2012 Aug 24;7(8):e43852. doi: 10.1371/journal.pone.0043852 (PMC3427222; doi:10.1371/journal.pone.0043852)
Supplement: Figure S2 — Diagrams of MES+HS treatment in vivo and in vitro and the experimental design. (PDF) [file pone.0043852.s002.pdf]

Figure S2.

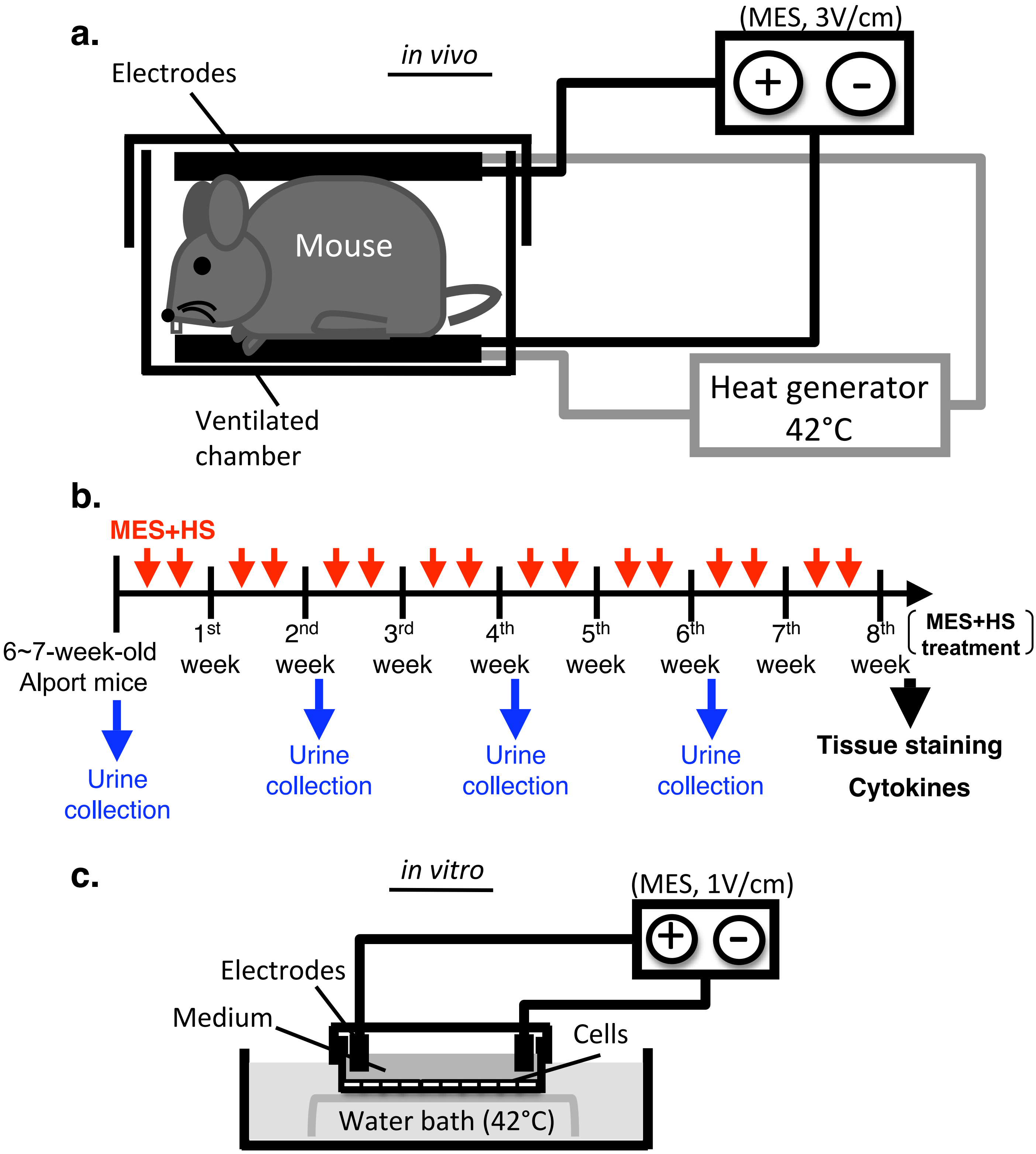

Figure S2. ***Diagrams of MES+HS treatment in vivo and in vitro and the experimental design.*** (*a*) Diagram of MES+HS treatment *in vivo*. (*b*) Schematic diagram of experimental design for MES+HS treatment *in vivo*. Six- to seven-week-old Alport mice were treated with MES+HS for 10 min twice a week. Urine samples were collected once per two weeks. Kidneys from Alport mice treated with MES+HS for 8 weeks were collected for analysis. (*c*) Diagram of MES+HS treatment *in vitro*.
