# Supplementary material for: Mild Electrical Stimulation and Heat Shock Ameliorates Progressive Proteinuria and Renal Inflammation in Mouse Model of Alport Syndrome
Source: PLoS One. 2012 Aug 24;7(8):e43852. doi: 10.1371/journal.pone.0043852 (PMC3427222; doi:10.1371/journal.pone.0043852)
Supplement: Figure S3 — MES+HS ameliorates glomerular apoptosis but has minimal effect on tubulointerstitial fibrosis. (PDF) [file pone.0043852.s003.pdf]

Figure S3.

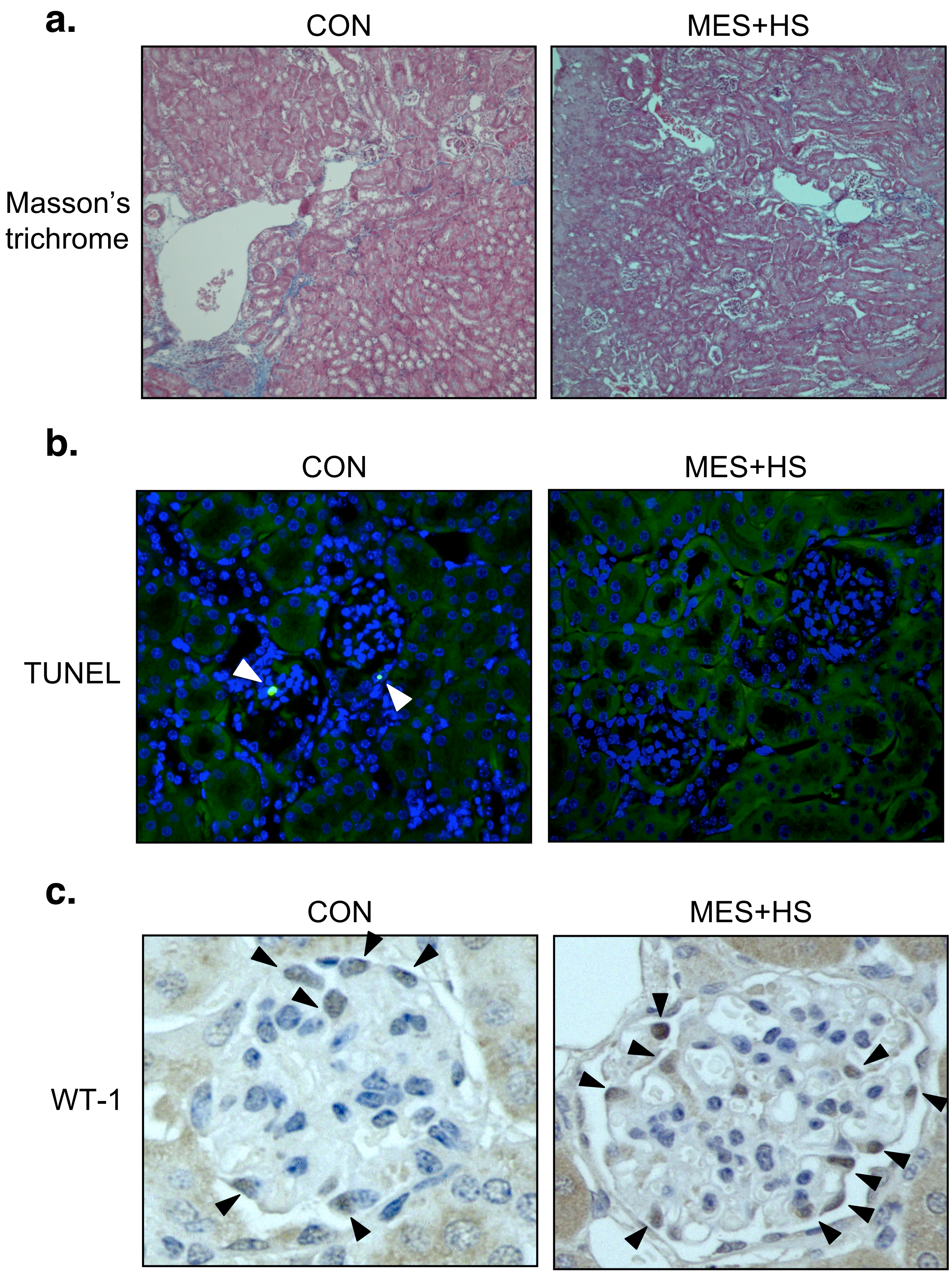

Figure S3. ***MES+HS ameliorates glomerular apoptosis but has minimal effect on tubulointerstitial fibrosis.*** (*a-c*) Representative images of glomeruli prepared from sham-treated (CON) or MES+HS-treated Alport mice. Tissue sections were stained with Masson's trichrome (*a*), TUNEL (*b*) and anti-WT-1 antibody (*c*). (*b, c*) Sections were counterstained with DAPI.
