# Supplementary material for: Mild Electrical Stimulation and Heat Shock Ameliorates Progressive Proteinuria and Renal Inflammation in Mouse Model of Alport Syndrome
Source: PLoS One. 2012 Aug 24;7(8):e43852. doi: 10.1371/journal.pone.0043852 (PMC3427222; doi:10.1371/journal.pone.0043852)
Supplement: Figure S4 — Inflammation-associated transcriptome analysis in kidneys of X-linked Alport syndrome mouse model. (PDF) [file pone.0043852.s004.pdf]

Figure S4.

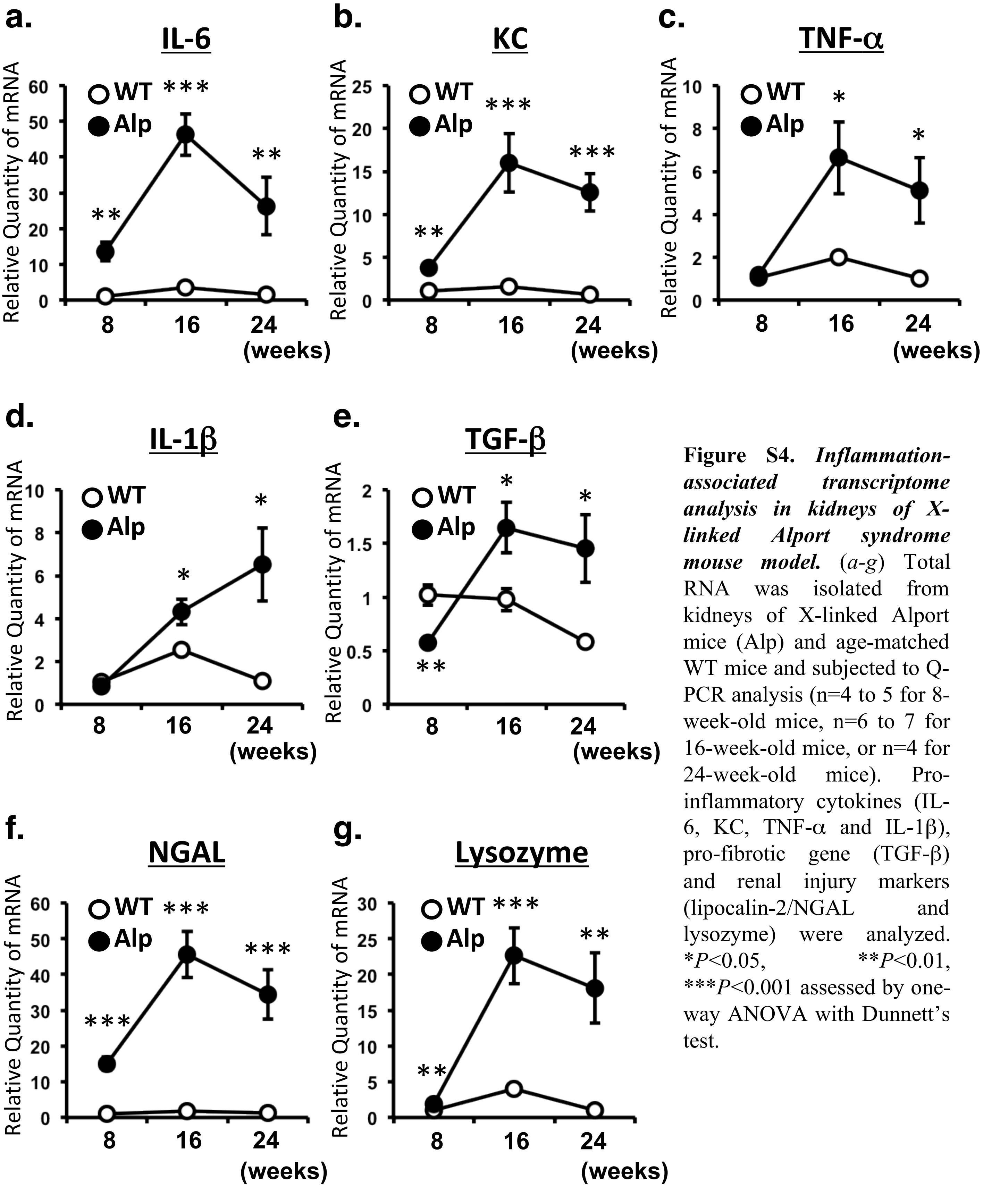

Figure S4. ***Inflammation-associated transcriptome analysis in kidneys of X-linked Alport syndrome mouse model.*** (*a-g*) Total RNA was isolated from kidneys of X-linked Alport mice (Alp) and age-matched WT mice and subjected to Q-PCR analysis (n=4 to 5 for 8-week-old mice, n=6 to 7 for 16-week-old mice, or n=4 for 24-week-old mice). Pro-inflammatory cytokines (IL-6, KC, TNF-α and IL-1β), pro-fibrotic gene (TGF-β) and renal injury markers (lipocalin-2/NGAL and lysozyme) were analyzed. *$P<0.05$, **$P<0.01$, ***$P<0.001$ assessed by one-way ANOVA with Dunnett's test.
