# Supplementary material for: Mild Electrical Stimulation and Heat Shock Ameliorates Progressive Proteinuria and Renal Inflammation in Mouse Model of Alport Syndrome
Source: PLoS One. 2012 Aug 24;7(8):e43852. doi: 10.1371/journal.pone.0043852 (PMC3427222; doi:10.1371/journal.pone.0043852)
Supplement: Figure S5 — MES+HS activates Akt through membrane cholesterol in podocyte in vitro and induces HO-1 mRNA expression in Alport glomeruli ex vivo. (PDF) [file pone.0043852.s005.pdf]

Figure S5.

a.

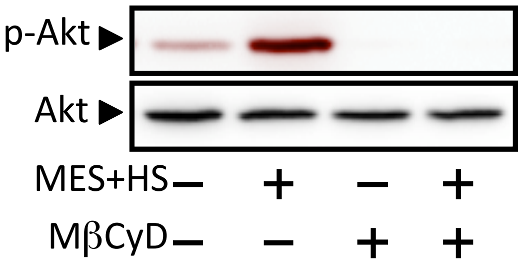

b.

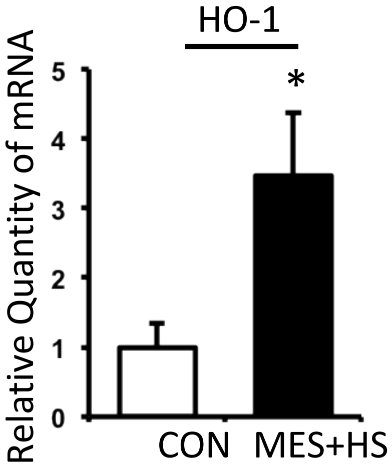

Figure S5. ***MES+HS activates Akt through membrane cholesterol in podocyte in vitro* and induces HO-1 mRNA expression in Alport glomeruli *ex vivo.*** (*a*) Podocytes were treated with MES+HS for 10 minutes with or without 5 mM methyl β cyclodextrin (MβCyD). Total cell lysates were analyzed by immunoblotting to check the phospho-Akt or total Akt protein expression. (*b*) Isolated Alport glomeruli were treated with MES+HS for 10 minutes and total RNA was extracted 5 hr after the treatment. Total RNA was subjected to Q-PCR analysis to check the mRNA expression of HO-1 (n=3). *$P<0.05$ as assessed by unpaired *t*-test.
