# Supplementary material for: Mild Electrical Stimulation and Heat Shock Ameliorates Progressive Proteinuria and Renal Inflammation in Mouse Model of Alport Syndrome
Source: PLoS One. 2012 Aug 24;7(8):e43852. doi: 10.1371/journal.pone.0043852 (PMC3427222; doi:10.1371/journal.pone.0043852)
Supplement: Figure S6 — MES+HS does not affect the survival of Alport mice. (PDF) [file pone.0043852.s006.pdf]

Figure S6.

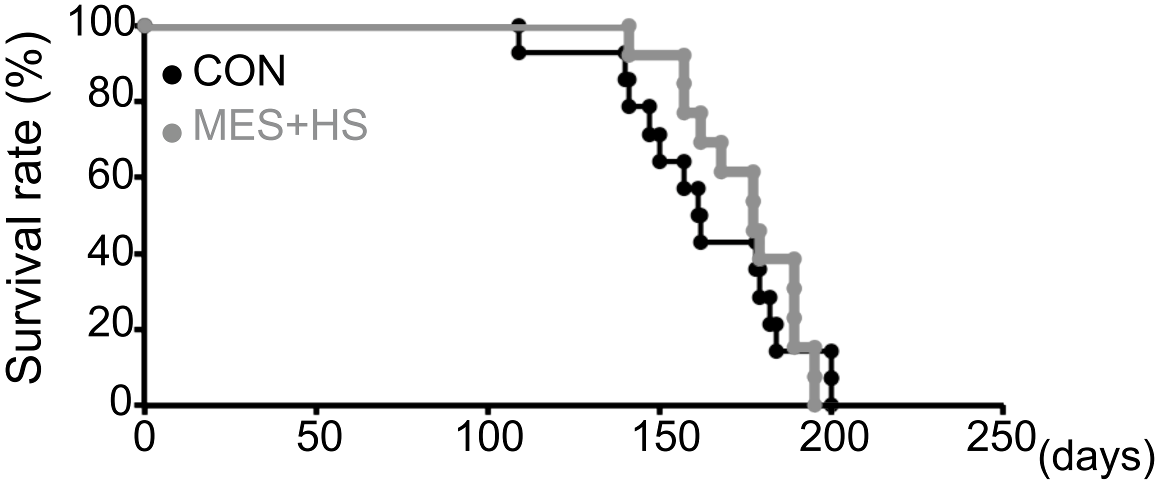

Figure S6. ***MES+HS does not affect the survival of Alport mice.*** Six- to seven-week-old Alport mice were treated with MES+HS for 10 min twice a week. Survival rate is illustrated as Kaplan-Meier curve (n=14 for each group). Black circle: sham-treated group (CON), grey circle: MES+HS-treated group (MES+HS).
